# The Lived Experience of Loneliness in Transgender Older Adults: A Qualitative Descriptive Study

**DOI:** 10.1093/geroni/igaf122.4027

**Published:** 2025-12-31

**Authors:** Katherine Bowers, Joan Carpenter

**Affiliations:** University of Maryland, Baltimore, Maryland, United States; University of Maryland School of Nursing, Baltimore, Maryland, United States

## Abstract

Loneliness affects more than a third of community dwelling older adults and is associated with poor physical and mental health, cognitive decline, and increased mortality. Transgender individuals experience health and socioeconomic disparities that place them at high risk for loneliness. The purpose of this study is to examine loneliness in transgender adults aged 50 and over and fill a critical knowledge gap within LGBTQIA+ aging research. Guided by the Health Equity Promotion Model, we conducted a qualitative descriptive study. Participants were sampled through gender affirming care centers in Maryland and local and national transgender support groups. Through semi-structured interviews and journaling, participants described their experiences with loneliness. Data were coded using conventional content analysis. Participants (N = 9) were 56-76 years old and had diverse gender identities. Three themes emerged regarding the lived experience of loneliness in transgender older adults: (1) loneliness on the inside; (2) longing for connection within relationships; (3) loneliness imposed by the outside world. The intersection of age and transgender identity heightens marginalization and elevates loneliness due to discrimination and social exclusion. Transitioning later in life, related to only recent access to gender affirming care, leads to complicated social challenges and partner strain increasing loneliness. Importantly, the current political climate has increased fear of persecution and discrimination; participants reported its tremendous impact on their isolation and loneliness. This study reveals that loneliness is prominent in transgender older adults. Given the unique vulnerabilities this population experiences, it is imperative that loneliness is addressed at both individual and societal levels.

